# Faster R-CNN and Geometric Transformation-Based Detection of Driver’s Eyes Using Multiple Near-Infrared Camera Sensors

**DOI:** 10.3390/s19010197

**Published:** 2019-01-07

**Authors:** Sung Ho Park, Hyo Sik Yoon, Kang Ryoung Park

**Affiliations:** Division of Electronics and Electrical Engineering, Dongguk University, 30 Pildong-ro 1-gil, Jung-gu, Seoul 100-715, Korea; pshgod91@dongguk.edu (S.H.P.); yoonhs@dongguk.edu (H.S.Y.)

**Keywords:** gaze tracking, driver’s eye detection, shallow CNN, faster R-CNN, geometric transformation

## Abstract

Studies are being actively conducted on camera-based driver gaze tracking in a vehicle environment for vehicle interfaces and analyzing forward attention for judging driver inattention. In existing studies on the single-camera-based method, there are frequent situations in which the eye information necessary for gaze tracking cannot be observed well in the camera input image owing to the turning of the driver’s head during driving. To solve this problem, existing studies have used multiple-camera-based methods to obtain images to track the driver’s gaze. However, this method has the drawback of an excessive computation process and processing time, as it involves detecting the eyes and extracting the features of all images obtained from multiple cameras. This makes it difficult to implement it in an actual vehicle environment. To solve these limitations of existing studies, this study proposes a method that uses a shallow convolutional neural network (CNN) for the images of the driver’s face acquired from two cameras to adaptively select camera images more suitable for detecting eye position; faster R-CNN is applied to the selected driver images, and after the driver’s eyes are detected, the eye positions of the camera image of the other side are mapped through a geometric transformation matrix. Experiments were conducted using the self-built Dongguk Dual Camera-based Driver Database (DDCD-DB1) including the images of 26 participants acquired from inside a vehicle and the Columbia Gaze Data Set (CAVE-DB) open database. The results confirmed that the performance of the proposed method is superior to those of the existing methods.

## 1. Introduction

Many traffic accidents occur owing to driver inattention, making this problem a severe issue in society. Drivers with reduced alertness are significantly less capable of controlling the vehicle, posing a serious risk to themselves and others. According to reported data, 35–50% of accidents are caused by factors related to insufficient driver attention such as distraction, fatigue, and drowsiness during driving. In the US in one year, 5870 people died and 515,000 were injured in accidents [1,2]. To solve these problems, studies are being actively conducted to confirm the driver’s forward attention and state. Technologies that detect the driver’s eyes to track gaze and judge the driver’s current state are the main study focus. The movements of the eyes provide valuable information about the driver’s alertness. If optical movements can be measured, then the driver’s drowsiness, carefulness, and attentiveness can be predicted [3]. Therefore, through precise detection of the driver’s eyes in images acquired through cameras, this method can prevent future accidents by predicting whether the driver is drowsy or paying proper forward attention. Accordingly, this study proposes a method to accurately extract the position of a driver’s eyes in a vehicle environment. The rest of this paper is structured as follows: Section 2 contains an analysis of existing works, and Section 3 describes the contributions of this study. Section 4 provides a detailed explanation of the proposed method. Section 5 describes the experimental results with analysis. Section 6 presents our conclusions.

## 2. Related Works

Existing studies related to eye detection can be largely divided into those concerning a desktop computer environment and those in a vehicle environment. The former can be divided into eye detection studies that involve the use of a wearable device [4,5,6,7] and those that use a non-wearable device [8,9,10,11]. For studies using a wearable device, the wearable device can consistently extract the user’s eye location as it follows rotations or changes in head location, given that the device moves with the user’s head. However, it is inconvenient in that the user has to directly wear the device to conduct experiments. Particularly in a vehicle environment in which the driver must drive for a long period of time, the inconvenience due to wearing the device is further worsened and it adds the risk of another distraction for the driver. Therefore, this method has limited application in an actual vehicle environment. Owing to these limitations, studies on eye detection in a vehicle environment mainly use non-wearable devices. In the non-wearable device eye detection method in a vehicle environment, a camera, usually installed inside the vehicle, obtains images of the driver’s face, and through these images, the driver’s eyes are either directly detected or detected from a limited search region after the face is detected. These methods can be largely divided into the single-camera-based method [12,13,14,15,16,17] and the multiple-camera-based method [18,19,20,21,22,23,24,25,26,27]. In the single-camera-based method, as the driver’s eyes are detected through the image information from a single camera, the complexity of the computation is relatively low. However, when the driver rotates his head, the eye region cannot be properly observed in the acquired image. Conversely, the multiple-camera-based method uses the combined image information of multiple cameras to detect the driver’s eyes, providing a high detection rate even when the driver rotates his head. However, this method has the drawback of high computation complexity. 

Vicente et al. proposed a driver inattention warning system using a single-camera-based method [13]. The researchers installed a camera and near-infrared (NIR) illuminator inside a vehicle and monitored the driver; the scale-invariant-feature-transform-based supervised descent method was applied to the obtained images. Accordingly, more accurate facial feature points of the driver could be detected, and the head pose and eyes of the 3D model could be finally detected. This method has the advantages of not being influenced significantly by the camera changing positions and having fast processing time when using the information obtained from the camera. However, it can be affected by external sources of light as an NIR illuminator cannot be used in experiments during the daytime, and the extraction of facial feature points is severely affected by the driver turning his head. Using a similar method, Fridman et al. detected the driver’s face and eyes through facial feature tracking [14]. These researchers investigated the effects of head pose and eye pose on categories of gazes in a vehicle environment. In the driver-monitoring images obtained through an in-vehicle single-camera-based method, a DLIB C++ library face detector [28] was used to detect the face and eyes; only 79.4% of the data was useful for face detection. In the study of [15,16], skin color learned beforehand was used for the skin color of the face to distinguish regions that were not skin as eyes. These researchers used the learned lip color of the driver in the image from the camera to detect the lip region of the driver, and thereafter used the learned color of the face to detect regions on the face of a different color, i.e., the eyes and lips. The eyes were determined as two regions above the lips, matching a certain size. In these detected eye regions, if eye white pixel is included, then it was judged as open eye, and otherwise, it was judged as closed eye, thereby forming a system that determines the state of the driver. The researchers used a single-camera-based method to avoid excessive computational cost. Consequently, it was vulnerable to problems caused by the driver turning his head during the detection process of eyes and lips. In addition, the distance between the driver and the installed camera was used as a condition in determining the size of the detected eye region. As a certain limited distance was used in the calculation, accurate eye detection could be difficult when the distance changed owing to the driver’s movements. They also could not solve limitations caused by wearers of glasses. In addition, Diddi et al. used the adaptive boosting (AdaBoost) method [29] to detect the face and eyes to determine the driver’s condition and provide a warning system. Images obtained from a single camera were used as input in AdaBoost with adaptive template matching to detect the face and eyes. The eye index (EI) was calculated from the detected eyes; it was classified into open, half-closed, and closed eyes, and was used for determining the driver’s state. While the adaptive-boosting-based method has the advantage of fast processing time, it is affected by external light through the visible light camera, and eye detection accuracy is affected by the driver turning his head. 

To overcome these disadvantages of the single-camera-based method, the multiple-camera-based methods have been studied. Existing studies [18,19] have proposed systems that check for driver inattention through two cameras installed in the vehicle. In particular, in [18], a faceLab eye-tracking system was used in the images of the driver obtained through the cameras installed on the dashboard and steering wheel to detect the user’s eyes. Through eye movement, a support vector machine (SVM) model was used to determine whether the driver was distracted. Ahlstrom et al. also used a commercial eye-tracking device for eye detection and proposed a system to detect driver inattention [20]. The driver’s face was monitored through two cameras installed behind the A-pillar and center console in the vehicle; SmartEye Pro 4.0, an eye-tracking device, was used to detect the eyes and measure the direction of the driver’s gaze. The images of the driver monitored through two cameras can effectively obtain data even in a wide range of head rotation. However, an expensive eye-tracking device was used to detect the eyes, and the performance of the proposed system depends on the performance of the eye-tracking device. In addition, the eye-tracking data of 23% of the total mileage in the experiment had low reliability. Moreover, to effectively detect the driver’s eyes, the experiment was conducted with restrictions on glasses, mascara, beards, moustaches, and other factors. Tawari et al. detected facial feature landmarks using a local-feature-based method in the driver images obtained through more than two cameras inside the vehicle [21]. Accordingly, they proposed a system to measure the state of the driver through eye and head pose detection [22]. Bergasa et al. proposed a system that analyzes the degree of driver inattention [23]; eyes are detected from the face model points inside the face region obtained from the AdaBoost face detector [30], and the percentage eye closure parameter is measured and combined with other visual cues. Ji et al. proposed a system that determines the state of the driver using various visual cues through eye detection [24]. The driver was monitored through two cameras installed on the dashboard inside the vehicle; one camera monitored the face through a wide angle focused on the face, whereas the other camera monitored the eyes through a narrow angle focused on the eyes. First, a two-ring NIR illuminator was used to obtain images of bright and dark pupils, and after detecting the pupil regions through the vehicle images of the two images, regions deemed eye candidates were distinguished using the SVM model to determine the actual eye regions. Based on the obtained eye information, the eyelid and gaze movement information was calculated and combined with other visual information obtained from the face images of the wide-angle camera, thereby determining the driver state and measuring inattention. The use of the NIR illuminator minimizes the influence of nearby lighting, securing image quality in various actual environments including day and night. Here, eye detection is simpler and faster than the process of eye detection following face detection. However, the experiment was not conducted in a real vehicle environment; the narrow-angle camera that focuses on the driver’s eyes requires a precise process to match the focus. In addition, as driver images from various angles cannot be obtained from the camera, it is vulnerable to excessive rotations of the driver’s head. When accurate bright or dark pupil images cannot be obtained (e.g., when the head is turned or is far from the camera), there is difficulty in eye detection. In addition, there have also been studies on eye detection algorithms that classify the region into “eyes” or “not eyes” using a neural classifier after eye location is detected through the Hough transform method [27]. This multiple-camera-based method allows free movement of the driver’s head and achieves high reliability through the combination of information from two images. The main drawback is the high computational cost of processing two images.

Accordingly, this study adopts the multiple-camera-based method, allowing highly reliable eye detection. This study also solves the problem of high computational cost to improve the performance of the existing eye detection method. Table 1 shows the summarized comparisons of the existing studies with the proposed method for eye detection in a vehicle environment.

## 3. Contributions

Compared with the previous works, our study is novel in the following four ways:

- This study is the first to apply a convolutional neural network (CNN)-based method in an in-vehicle multiple-camera-based environment to attempt to detect the driver’s eyes.
- After composing a three-channel image from the two driver face images obtained using the two cameras from various angles, the image is used as input in the shallow CNN to select a frontal face image. In the selected frontal face image, the eyes barely ever disappear or are covered owing to head rotations of the driver, showing more effective eye detection results
- For the two driver images obtained through the two cameras, faster R-CNN is applied to the frontal face image only rather than to both, and the eye positions of the other image are detected through geometric transformation, thereby preserving eye detection accuracy while reducing processing time.
- The self-built Dongguk Dual Camera-based Driver Database (DDCD-DB1), learned faster R-CNN, and algorithms are released as shown in [31] in order to enable other researchers to perform a fair performance evaluation.

## 4. Proposed System for Detection of Driver’s Eyes in Vehicle Environments

### 4.1. Overview of Proposed Method

**Figure 1:**
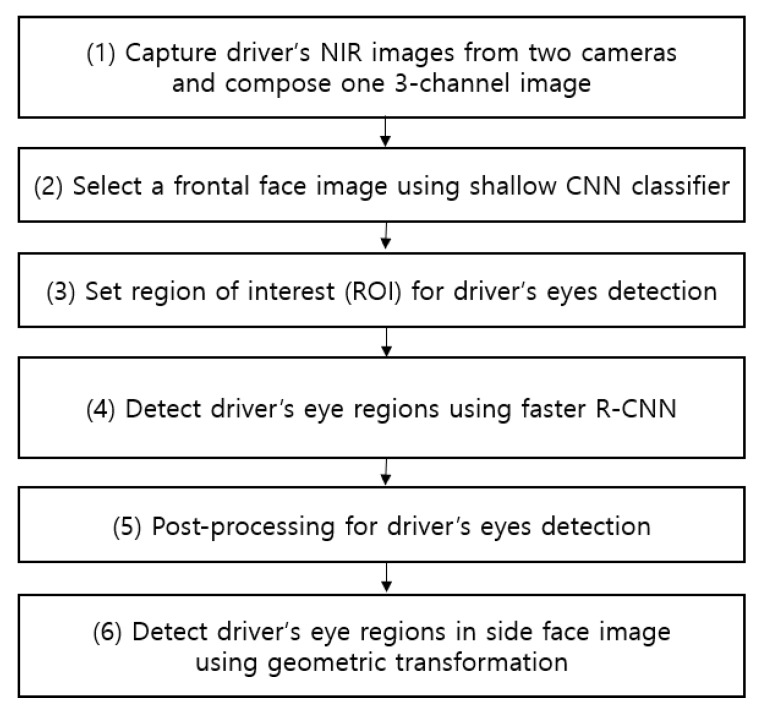
Flowchart of the proposed system.

Figure 1 shows an overall flowchart related to the proposed detection method for the driver’s eyes.

**Figure 2:**
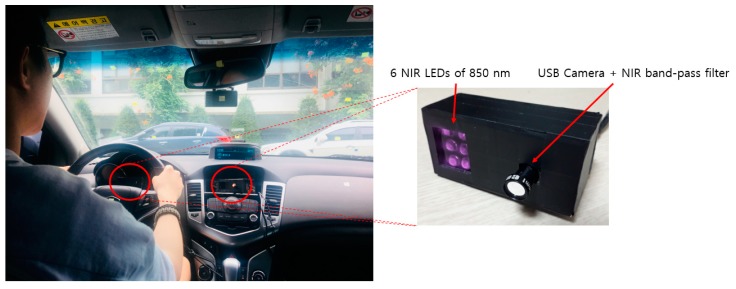
Experimental setup in the vehicle environment.

As shown in Figure 2, two image-capturing devices containing an illuminator composed of an NIR camera and six NIR light-emitting diodes (LEDs) of 850 nm are installed, one each at the front of the vehicle dashboard and the center of the vehicle, to monitor the driver. As the 850 nm NIR illuminator used in this study has nearly no glare, it does not interfere with the driver’s vision or influence driving. It enables images to be obtained at a brightness at which the driver’s eyes can be detected regardless of external lighting during the day and night. By using the NIR cameras equipped with this illuminator, it is possible to monitor the driver from wide angles without being affected by external lighting. As shown in Figure 2, an 850 nm band-pass filter is attached to the NIR camera to minimize the external influences such as sunlight. We could thus obtain images with uniform brightness relying only on the 850 nm NIR used in this study. These two image-capturing devices were used to obtain driver images of 1600 × 1200 in pixel size (three channels), and through the two images, a three-channel composite image was generated (Step (1) of Figure 1). The composite image obtained thus was used as input in the shallow CNN to select an image closer to the frontal face (Step (2) of Figure 1, and the details are explained in Section 4.2). This is because, in the side face image, a part of the eye disappears or is covered owing to the head being turned, making eye detection difficult. However, the use of a frontal face image provides a higher eye detection rate. To detect eyes more efficiently from the frontal face image, the region of interest (ROI) of the eye region is set (Step (3) of Figure 1). The ROI set thus is used as input in the faster R-CNN, enabling more accurate eye detection (Step (4) of Figure 1, and the details are explained in Section 4.3). Among the eye candidates obtained through faster R-CNN, the distance and angle conditions among the candidate regions are applied and the candidate regions outside this range are deemed erroneous detections and are excluded. Subsequently, the final two eye detection candidate regions are selected (Step (5) of Figure 1, and the details are explained in Section 4.4). A geometric transformation matrix is used for the two cameras and the final eye regions obtained through faster R-CNN from the frontal face image. The eye position of the remaining side face image is detected (Step (6) of Figure 1 and the details are explained in Section 4.5). 

### 4.2. Classification of Frontal Face Image Using Shallow CNN

**Figure 3:**
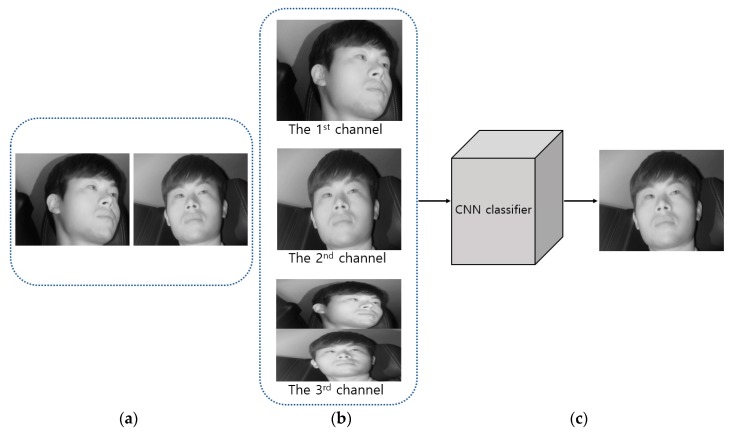
Classification of frontal face image. (**a**) Image captured through the 1st camera and the 2nd camera; (**b**) Combined three-channel image for the input to shallow CNN; (**c**) Selected frontal face image.

**Figure 4:**
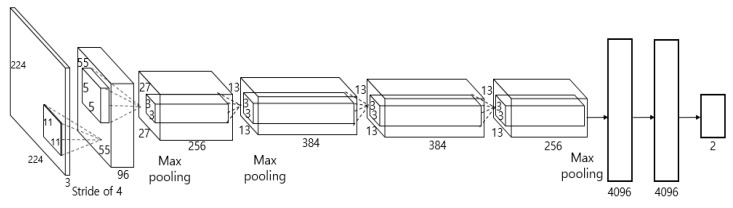
Structure of the shallow CNN.

As described in step (2) of Figure 1, the images obtained from two cameras are used as input in shallow CNN and the image closer to the frontal face is selected, as shown in Figure 3. Figure 4 shows the CNN structure used in this study; the AlexNet model was used through the CNN model [32]. The AlexNet model used is a model pre-trained through the ImageNet database [33]. It was fine-tuned through the training data used in this study to classify the frontal face image. 

As shown in Figure 2, the images obtained through the first and second cameras are included as the 1st and 2nd channel images. In addition, the two images obtained through the first and second cameras are squeezed vertically, and the image included in the upper and lower parts of an image is included as the 3rd channel image. This three-channel image is used as input in the shallow CNN.

As shown in Figure 4, AlexNet used as the shallow CNN has a relatively shallow structure of five convolutional layers, three pooling layers, and three fully connected layers. Unlike other CNN models formed from complex structures, by using the AlexNet model, this study can classify the frontal face image even faster [34]. 

By fine-tuning the pre-trained AlexNet model, the used input images were resized into 224 × 224 pixels through bilinear interpolation. The training method for AlexNet fine-tuning is described in Section 5.2.1. Each of the five convolutional layers has kernels of different sizes that extract the features of the input, and after passing through all five convolutional layers, five rectified linear unit (ReLU) layers [35], and three max pooling layers, a feature map of size 13 × 13 × 256 is generated. The size of the feature map is calculated based on the method of [36]. The output nodes of the first and second layers of the fully connected layer, the step for classification through the generated feature map, include 4096 nodes. The nodes of the third and final fully connected layer include two output nodes, so that, if the images obtained from the first and second cameras are close to the frontal face, then the image can be classified into two classes. This study uses this shallow CNN model to classify the driver frontal face image faster and more accurately. The classified frontal face image is used as input in the faster R-CNN for eye detection, achieving higher performance.

### 4.3. Eye Detection Using Faster R-CNN

#### 4.3.1. Structure of Faster R-CNN

**Figure 5:**
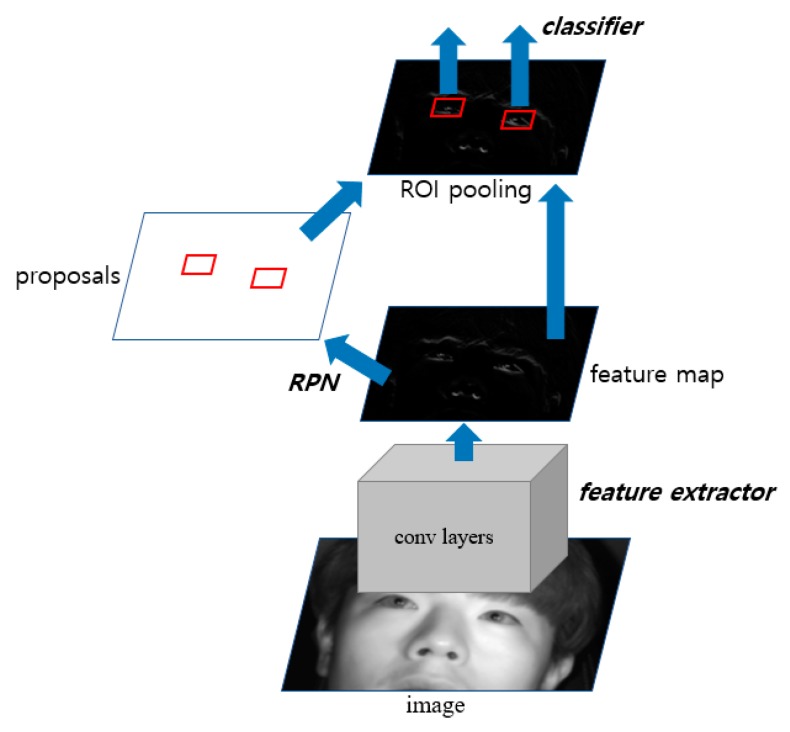
The structure of faster R-CNN.

The image selected through the frontal face image using the shallow CNN is used as input to faster R-CNN [37], and the eye region of the driver is detected. The structure of the faster R-CNN used is shown in Figure 5. It is largely divided into three parts: the feature extractor, region proposal networks (RPN), and classifier. First, to take a simple look at the process of faster R-CNN, the feature map is created after passing through the last convolutional layer in the feature extractor and is used as input in the RPN. Subsequently, the proposals of the object to be detected are generated. The generated proposals enter the fully connected layer as input and are classified by each class, and subsequently, a score for them is determined. Table 2, Table 3 and Table 4 describe the structure in detail.

**Figure 6:**
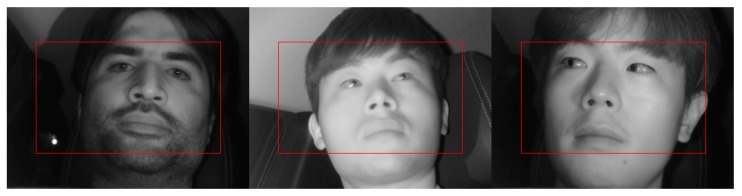
Examples of ROI for the input to faster R-CNN.

Table 2 shows the structure of the feature extractor used in this study. We used a pre-trained VGG Net-16 network [38] through ImageNet [33] as a feature extractor model. In the structure of the VGG Net-16 network, the structure of the network up to before the final max pooling layer was used to extract the features. This structure consists of 13 convolutional layers, 13 ReLU layers, and four max pooling layers. First, the classified frontal face image is received as input and passed through the convolutional layers, ReLU layers, and max pooling layers to obtain the feature maps for the final input image. In the input image of 1600 × 1200 (width × height) pixels, ROI is set for the region around the eyes within the entire face, allowing the driver’s eyes to be more effectively detected. Based on the training data used in the experiment, the ROI region is set considering various changing positions owing to head rotation during driving. As shown in Figure 6, the ROI of 1400 × 800 is determined based on the first 1600 × 1200 input image and cropped to minimize the region that can be designated as eye candidates, thereby increasing the detection rate. The final feature maps of size 88 × 50 × 512 pixels (width × height × channel) are obtained from the in-vehicle driver image of size 1400 × 800 pixels. The feature maps obtained here are used later in the RPN and classifier as input.

Table 3 shows the structure of the RPN used in this study. The RPN is designed to efficiently provide a region proposal of various sizes and ratios for the object using the last feature map obtained in the previous feature extractor step. This has a fully convolutional network structure. It obtains anchor boxes of nine sizes and ratios for the image of the position corresponding to the feature map through the last 1 × 1 convolutional layer. In addition, the driver’s eyes probability and bounding box regression vector [39] are obtained for the anchor boxes to effectively provide an eyes proposal region. We reduce the scale of the anchor boxes used in the existing faster R-CNN [37] to set anchor boxes of a size more suitable for eye detection, enabling more accurate detection. The following Equations (1) and (2) are bounding box regression vectors ($v_{x},\;v_{y},\;v_{w},\;v_{h}$), which are parameterized values of the transformation between the anchor box and the predicted box [39]:

(1) $$v_{x}=\frac{x_{p}-x_{a}}{w_{a}},\;v_{y}=\frac{y_{p}-y_{a}}{h_{a}}$$

(2) $$v_{w}=\log\left(\frac{w_{p}}{w_{a}}\right),\;v_{h}=\log\left(\frac{h_{p}}{h_{a}}\right)$$

In Equations (1) and (2), (x, y, w, h) indicates the center coordinates x, y and width and height of each box, respectively. In addition, $x_{p},\;x_{a}$ indicate the center coordinate *x* of each proposal box and anchor box, respectively (The same applies to y, w, and h). Equation (1) shows the scale-invariant translation between the center coordinates, whereas Equation (2) shows the log-space translation between the width and height. Through this obtained bounding box regression vector, a proposal box translated with more suitable scale and location can be obtained. The final obtained proposal boxes correspond to the intersection over union (IOU) threshold (i.e., IOU > 0.7); non-maximum suppression (NMS) is conducted to select N (i.e., 300) proposal boxes above the eye scores (i.e., object probability) standard to obtain the eye candidates. 

Table 4 shows the structure of the classifier used in this study. In this step, the final feature map and proposal boxes obtained in the above two steps are used as input. First, after cropping the corresponding location to the proposal boxes on the feature map, it passes through the fully connected layer, and proposal boxes of different sizes are adjusted to the same size (i.e., 7 × 7) through ROI pooling. After this feature map with adjusted size passes through the fully connected layer, the bounding box regression vector and eye scores are obtained, and re-refined suitable predict boxes are obtained from the box regression vector. NMS is used to remove overlapping candidate boxes, thereby obtaining the final detection results. As the existing faster R-CNN was designed for multi-class detection (i.e., 20 classes), each proposal is classified as multi-class in the classifier section. In this study, to classify the results into three classes (i.e., open eye, closed eye, and background), the output nodes are reduced to three in the classification step and used (in Table 4, 3 indicates the probabilities of three classes i.e., open eye, closed eye, and background, and 300 indicates the number of candidates.)

#### 4.3.2. Loss Function

In this study, faster R-CNN performs classification of two classes (object and background) in RPN and classification of three classes (open eye, closed eye, background) in classifier. Thus, in each RPN and classifier structure, weight is trained to minimize the loss function for each anchor box or proposal box in the mini-batch:

(3) $$L\left(p,p^{*},v,v^{*}\right)=L_{cls}\left(p,p^{*}\right)+\sigma p^{*}L_{reg}\left(v,v^{*}\right)$$

Equation (3) above shows the loss function used in the RPN and classifier structures. First, p in the RPN indicates the probability that the anchor box is the object. $p^{*}$ indicates the ground-truth label (object = 1, background = 0), v indicates the bounding-box regression vector of the anchor box, and $v^{*}$ is the bounding-box regression vector of the anchor box and related ground-truth. In the classifier structure, p indicates the probability distribution corresponding to each predict box (for each predict box, p = ($p_{0},p_{1},p_{2}$)). $p^{*}$ indicates the ground-truth label (open eye = 1, closed eye = 2, background = 0), and Equation (3) above becomes 1 when $p^{*}$ is not the background label. v indicates the corresponding bounding-box regression vector of each class of $p^{*}$. $v^{*}$ indicates the bounding-box regression vector of the corresponding class and related ground-truth. $L_{cls}$ (classification loss function) and $L_{reg}$ (regression loss function) each correspond to log loss function and robust loss function (smooth $L_{1}$) [40]. In Equation (3), regression loss occurs only when ground truth is not the background ($p^{*}\neq$ 0). Therefore, when the ground truth is open eye or closed eye, classification loss and regression loss are combined and calculated; when it is background, only classification loss is calculated and minimized. Finally, cls, reg are weighted with a weight balancing parameter σ. Progressing through this process, weight is trained to minimize the loss value and the eye position is detected in the driver image. An operation is conducted to determine whether the detected eye is open or closed.

### 4.4. Post-Processing for Eyes Detection

**Figure 7:**
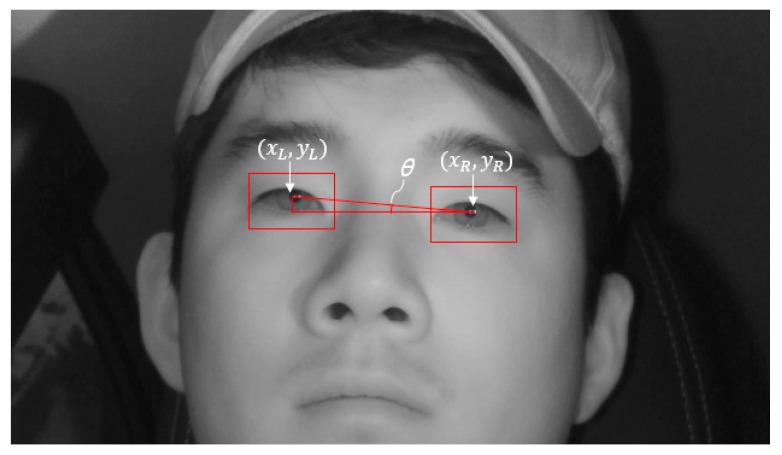
An example of measuring *θ* using the center coordinates of left and right eye boxes.

In the process of detecting the driver’s eyes through faster R-CNN described above, we inserted several detection conditions to raise the rate of detection. The number of driver’s eyes is fixed at two, so that, if there are three or more final eye detection candidate boxes, then the boxes other than the boxes for the two actual detected eye regions are judged as erroneously detected. Consequently, the post-processing conditions are applied and, after excluding the erroneously detected boxes, the final two eye detection boxes are selected. Normally when driving, the driver looks forward and to the side, and hence, the head roll angle does not extend beyond a certain range. Therefore, if there are three or more eye detection boxes, we first calculate the angle *θ*, the head roll angle between the boxes; only boxes that form angles within a certain range are selected. To calculate *θ*, we obtained center coordinates x and y of each detected box to calculate the distance between the detected boxes. $\left(x_{L},\;y_{L}\right)$ and $\left(x_{R},\;y_{R}\right)$ in Figure 7 indicate the center coordinates of the left eye detection box and right eye detection box, respectively, whereas *θ* indicates the angle between the eye boxes owing to head roll. The angle between the boxes, *θ*, is calculated through Equation (4). After calculating the angle between the boxes, *θ*, the two candidates that form the smallest of these values are first selected as eye boxes:

(4) $$\theta =\tan{}^{-1}\left(\frac{y_{R}-y_{L}}{x_{R}-x_{L}}\right)$$

Additionally, if the horizontal distance between the two selected boxes satisfies a specified distance condition, then they are selected as the final eye boxes. Here, the specified distance condition is set based on the horizontal distance between the actual two eyes in the input image based on the training data. If the two boxes that form the smallest *θ* value deviate from the specified distance (if the two eye candidates are too far or too close), then the two boxes that form the following narrower angle are selected as the eye candidate boxes, and the conditions are checked to determine whether they are satisfied. If the conditions are satisfied, only the two boxes are finally selected as the eyes detection results.

### 4.5. Detect Driver’s Eyes in Side Face Image Using Geometric Transform Matrix

**Figure 8:**
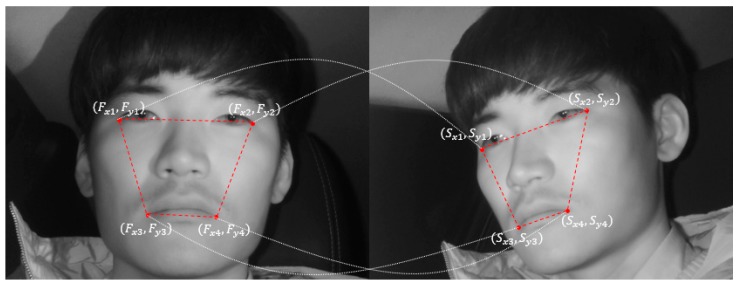
Mapping of eye regions using geometric transform.

The abovementioned faster R-CNN is applied to the images obtained from the two cameras, thus achieving high reliability. However, this method has the limitation of high computational cost. Therefore, this study uses geometric transformation [41] between the two cameras, as shown in Equations (5) and (6). The driver’s eyes of the frontal face image detected above are used to map the driver’s eye regions of the remaining images. Figure 8 shows the mapping of the driver’s eye regions between the frontal face image and side face image.

(5) $$S=T\cdot F\;\left[\begin{array}{cccc} S_{x1} & S_{x2} & S_{x3} & S_{x4}\\ S_{y1} & S_{y2} & S_{y3} & S_{y4} \end{array}\right]=\left[\begin{array}{cccc} a & b & c & d\\ e & f & g & h \end{array}\right]\left[\begin{array}{cccc} F_{x1} & F_{x2} & F_{x3} & F_{x4}\\ F_{y1} & F_{y2} & F_{y3} & F_{y4}\\ F_{x1}F_{y1} & F_{x2}F_{y2} & F_{x3}F_{y4} & F_{x4}F_{y4}\\ 1 & 1 & 1 & 1 \end{array}\right]$$

In Equation (5), the transformation matrix T is obtained by multiplying ***S*** and ***F^−1^***, and the geometric relationship between the frontal face image and side face image can be parameterized. The ***T*** matrix was obtained beforehand through training data. To solve the problem of ***T*** matrix differing by Z distance from the camera to the driver’s face, the ***T*** matrix is obtained beforehand for approximately three distances based on the distance between the driver’s eyes. The ***T*** matrix that matches the distance between the eyes of the input image is used. This obtained transformation matrix ***T*** and the driver’s eye box coordinates ($E_{f}$) from the frontal face image obtained in the abovementioned faster R-CNN are used to calculate the eye box coordinates ($E_{s}$) of the side face image, using Equation (6):

(6) $$E_{s}=T\cdot E_{f}\;\left[\begin{array}{cc} e_{sx1} & e_{sx2}\\ e_{sy1} & e_{sy2} \end{array}\right]=\left[\begin{array}{cccc} a & b & c & d\\ e & f & g & h \end{array}\right]\left[\begin{array}{cc} e_{fx1} & e_{fx2}\\ e_{fy1} & e_{fy2}\\ e_{fx1}e_{fy1} & e_{fx2}e_{fy2}\\ 1 & 1 \end{array}\right]$$

**Figure 9:**
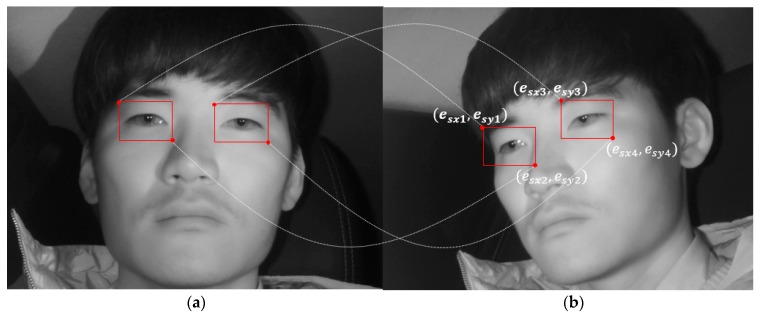
Mapping of eye regions in side face image. (**a**) Detecting eyes in frontal face image using faster R-CNN; (**b**) Mapping of eye regions in side face image using geometric transform.

In Equation (6), ($e_{fx1}$, $e_{fy1}$) and ($e_{fx2}$, $e_{fy2}$) indicate the left upper and right lower coordinates of the eye box from the frontal face image detected through faster R-CNN, respectively. ($e_{sx1}$, $e_{sy1}$) and ($e_{sx2},\;e_{sy2}$) indicate the left upper coordinates and right lower coordinates of the eye box to be mapped to each side face image, respectively. Accordingly, Equation (6) above is applied to both the left and right eyes detected through faster R-CNN (Figure 9a), and the left and right eye regions are both mapped in the side face image (Figure 9b). By applying geometric transformation thus, the driver’s eyes are finally detected in the images obtained from the two cameras.

## 5. Experimental Results with Analysis

### 5.1. Experimental Environment

**Figure 10:**
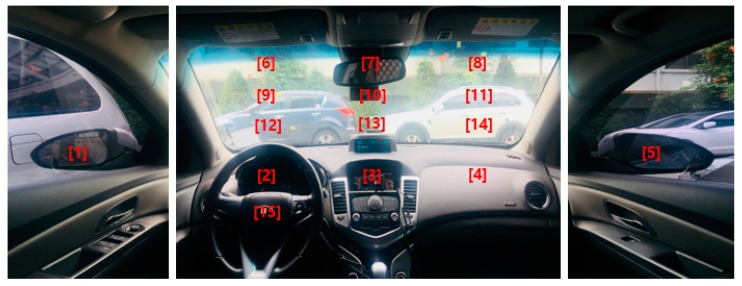
15 gaze zones in our experiments.

**Figure 11:**
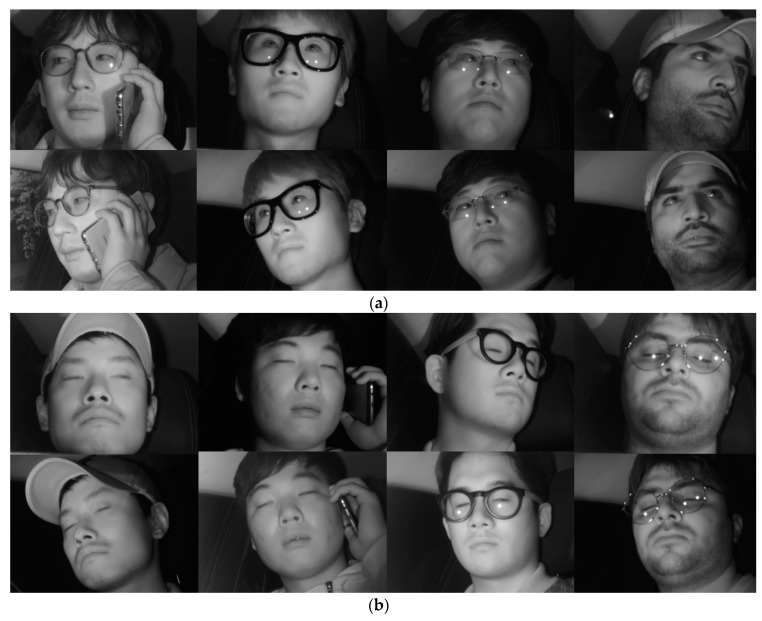
Examples of images in DDCD-DB1. (**a**) Driver’s open eye images captured through the 1st camera (the 1st row images) and 2nd camera (the 2nd row images); (**b**) Driver’s close eye images captured through the 1st camera (the 1st row images) and 2nd camera (the 2nd row images).

Most previous driver databases of vehicle environments are not open databases. Among existing databases, the Chinese Academy of Sciences Pose, Expression, Accessories, and Lighting (CAS-PEAL) face database [42] consists of data obtained in various poses, expressions, accessories, and lighting environments, and is thus widely used in facial recognition technology. However, as it is obtained in a laboratory rather than actual vehicle environments using a driver as the target, the database does not reflect the various elements of a vehicle environment. In addition, while there is another dataset with driver images in the RobeSafe driver monitoring video (RS-DMV) vehicle environment [43], as this dataset only contains data obtained from a single camera, it cannot be used in studies based on two cameras, such as this study. Consequently, to evaluate the performance of the system proposed in this study, we collected data for our own database (DDCD-DB1) through the experimental setup shown in Figure 2. DDCD-DB1, the learned faster R-CNN, and algorithms are released as shown in [31] in order to enable other researchers to perform a fair performance valuation. When acquiring DDCD-DB1, the driver’s gaze area was divided into 15 zones as shown in Figure 10. The drivers gazed at the 15 zones divided out beforehand in order, and a total of 26 participants were each assigned eight different situations (i.e., wearing a hat, wearing four different types of glasses (rimless, gold-rimmed, half-frame, and horn-rimmed), wearing sunglasses, making a call through mobile phone, covering face through hand, etc.), and the data were collected (Figure 11). By considering many different situations that could occur in actual driving, we could measure performance for various situations and obtain high reliability. As the participants gazed at the designated regions in turn, natural head rotations that would occur in actual driving were permitted, and other restrictions or instructions were not provided. When acquiring actual driving data, to avoid any risk of a traffic accident, rather than actually driving, a real vehicle (a Renault-Samsung model SM5 [44]) was started from a parked state in various locations (from daylight road to a parking garage). By conducting the experiments thus, data could be collected in an environment most similar to an actual driving situation. For all training and testing of the CNN model used in this study, an Intel^®^ Core™ i7-7700 CPU@3.60 GHz (Intel Corp., Santa Clara, CA, USA), 16 GB memory and NVIDIA GeForce GTX 1070 (1920 CUDA cores and 8 GB memory) graphics card [45] desktop computer was used. The CNN model training and testing algorithm used in this study was implemented through Windows Caffe (Version 1) [46].

All the experiments in our researches were performed based on two-fold cross validations. In detail, the data of half people (13 people) were randomly selected for training, and the remained data of the other 13 people were used for testing. Then, the data for training and testing were exchanged each other, and training and testing were repeated again. From this scheme, we obtained two testing accuracies and the average one was determined as final accuracy.

### 5.2. Performance Evaluation of the Classification of Frontal Face Image Using Shallow CNN

#### 5.2.1. Experimental Data and Training of Shallow CNN

**Figure 12:**
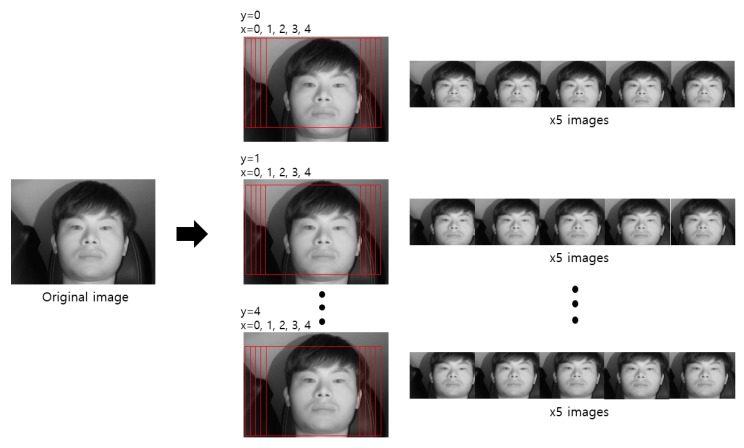
Examples of data augmentation through pixel shifting and cropping.

We trained the shallow CNN classifier to select the frontal face image among the images obtained from the two cameras for which the eyes almost never disappeared or were covered owing to head rotations. Table 5 shows the database used in this CNN model training and testing. To avoid the overfitting problem that may have occurred during the training process in this study, data augmentation was performed only for training data. For an image, a total of four pixels are each shifted one pixel up, down, left, and right, and 25 cropped augmented images were obtained (Figure 12). As shown in Table 5, the augmented images were only applied to training data; for testing data, original data rather than augmented images were used.

In this study, the stochastic gradient descent (SGD) method [47] was used in training the shallow CNN model. This is a method to determine the optimized weight that can minimize the difference between the result obtained from the CNN during training and the ground-truth result. Instead of all the data, a division of the training set via mini-batch size iterations method was used to conduct training at a faster speed with several iterations. In this study, the following parameters were used in performing the SGD method: base learning late = 0.0001, gamma = 0.1, batch size = 30, momentum = 0.9, weight decay = 0.0005, and epoch = 5. For the ground-truth frontal face image which has relatively fewer loss of eye area between two camera images, the image closer to the frontal face was manually selected. If both images had visible eyes and small head rotation and were close to the frontal face, then the image obtained from the first camera was classified as the frontal face image. 

**Figure 13:**
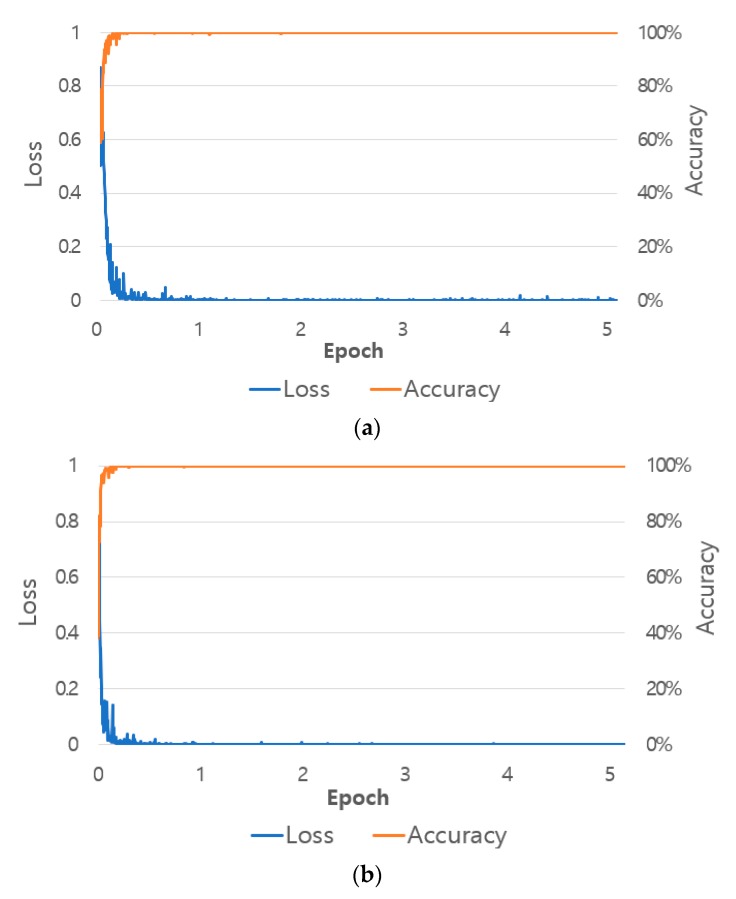
Training accuracy and loss graphs with shallow CNN in (**a**) the 1st fold validation, and (**b**) the 2nd fold validation.

Figure 13 shows a graph of loss and accuracy during training. The *x*-axis shows the number of performed epochs, the left side of the *y*-axis shows the loss value, and the right side of the *y*-axis shows training accuracy. As the number of epochs increases, loss converges closer to 0, and accuracy converges to 100%. This indicates that the shallow CNN training performed in this study successfully had no overfitting.

#### 5.2.2. Classification Accuracy with Shallow CNN

Through the aforementioned trained shallow CNN model, the frontal face image classification accuracy was measured, as shown in Table 6. As described, accuracy was measured through two-fold cross validation. The classified frontal face image is used as input in faster R-CNN for eye detection in Section 5.3. As shown in Table 6, we confirmed that the frontal face image was classified with an accuracy of at least 99.7% through the methods used in this study. The number of parameters in AlexNet is much larger than that in ResNet [32,48]. As shown in previous research [34], however, the processing speed by AlexNet is faster than that by ResNet, and the power consumption by AlexNet is also lower than that by ResNet because the number of floating point operations (FLOPs) by ResNet is larger than that by AlexNet [48,49]. From our experiment in the desktop computer whose specifications are explained in Section 5.1, the average processing time of one image by AlexNet was 3.58 ms whereas those by ResNet-18 and 34 [48] were 8.74 ms and 17.49 ms, respectively. Considering the case of applying our method to the embedded system having low processing power in actual car environment, we select AlexNet because of the lower processing time and power consumption although the number of parameters in AlexNet is much larger than ResNet.

**Figure 14:**
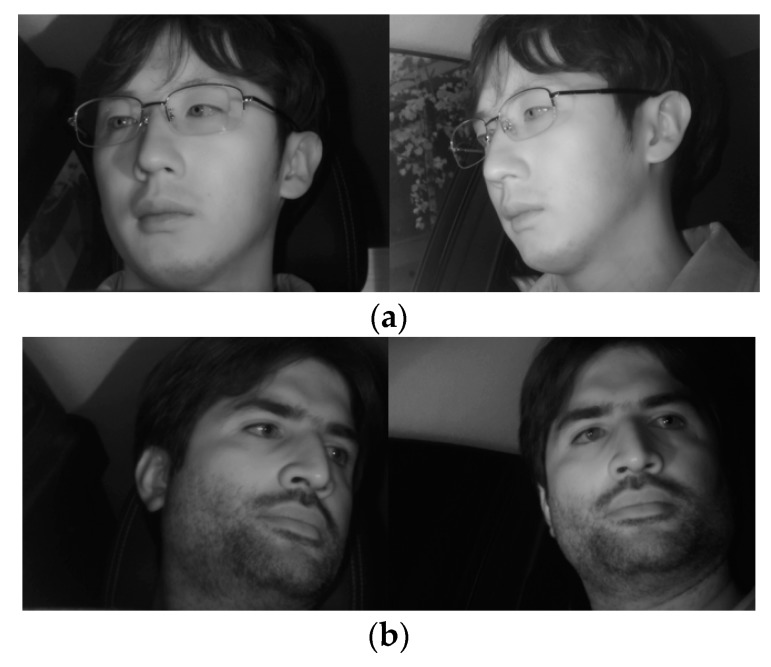
Examples of correct classification cases of frontal face image. (**a**) Left image selected; (**b**) Right image selected.

**Figure 15:**
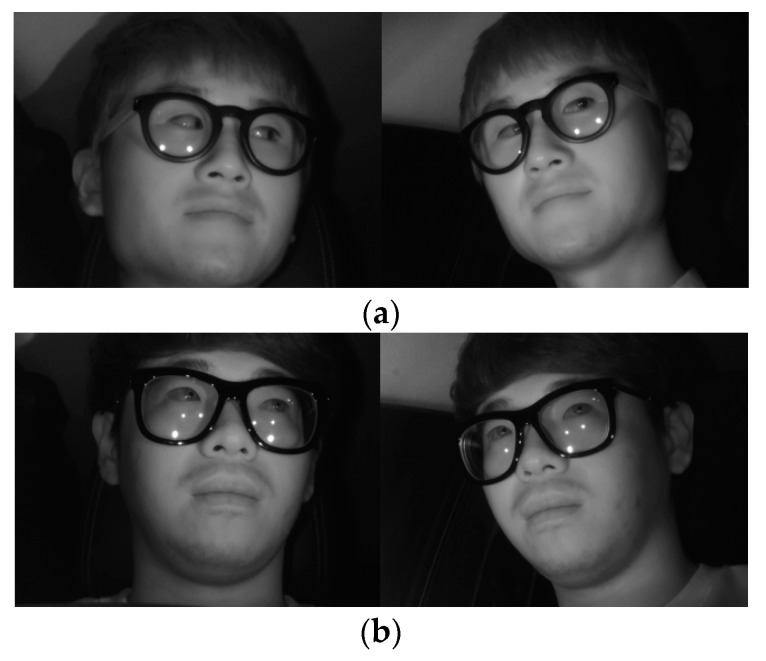
Incorrectly classified cases of frontal face image. (**a**) Right image selected; (**b**) Right image selected.

Figure 14 shows the correctly classified cases, whereas Figure 15 shows the error cases. As shown in Figure 14, the frontal face image was correctly classified regardless of whether glasses were worn. In the case of Figure 15, while the classification results seemed incorrect, both images were judged to be close to the frontal face; therefore, using them as the input images of faster R-CNN for the final eye detection is appropriate and would not have a significant effect on eye detection performance.

### 5.3. Performance Evaluation of Eye Detection

#### 5.3.1. Experimental Data and Training of Faster R-CNN

**Figure 16:**
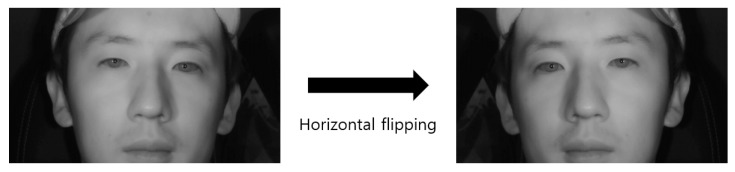
Data augmentation through horizontal flipping.

For the second experiment, the performance of the eye detection method proposed in this study was evaluated. Table 7 shows the database used in the training and testing of the faster R-CNN in this study. To classify open eye and closed eye while simultaneously detecting eyes, training was conducted using the labeled dataset for each class. A two-fold cross validation was performed for the performance evaluation. In addition, for faster R-CNN training robust in a larger variety of environmental changes, blurred images were added to the training data of Table 7, and the images were used as training data. Additionally, to avoid the overfitting problem, data augmentation was performed through horizontal flipping (Figure 16). Thus, twice the number of augmented images for training was obtained. As shown in Table 7, the augmented images were used only in the training data; for testing data, the original testing data of Table 7 were used.

**Figure 17:**
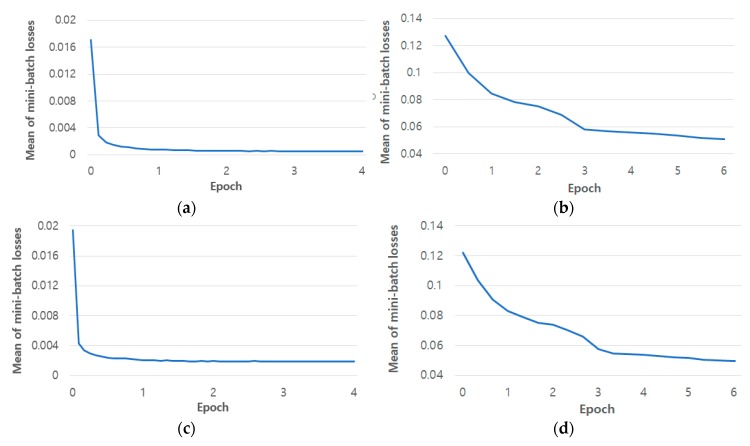
Loss graphs during faster R-CNN training in two-fold cross validation. (**a**) RPN losses from the 1st fold validation; (**b**) classifier losses from the 1st fold validation; (**c**) RPN losses from the 2nd fold validation; (**d**) classifier losses from the 2nd fold validation.

In the existing faster R-CNN, RPN learning and classifier learning were alternately performed. The four-step alternating training method was used, in which each process is performed twice [37]. In this study as well, training was conducted through this method. In the first training step of the RPN and classifier, end-to-end learning is conducted together with the feature extractor. In the second step of RPN and classifier training, to share the feature extractor, the feature extractor is excluded and only network learning is conducted. The SGD method was used during training with the following parameters: base learning late = 0.001, gamma = 0.1, batch size = 1, momentum = 0.9, weight decay = 0.0005, and epoch = 4 (in RPN training); base learning late = 0.001, gamma = 0.1, batch size = 2, momentum = 0.9, weight decay = 0.0005, and epoch = 6 (in classifier training). In each step during training, with each iteration interval (i.e., 2000), the trained model up to that point was saved, and after training, the model with the fewest validation errors among the saved models was selected and used in the next training step. Approximately two days were required to complete all the training steps. Figure 17 is a graph of loss of the final RPN and classifier training step. The *x*-axis represents the number of epochs and the *y*-axis represents the loss value. As epoch increases, loss converges toward a sufficiently low value. This indicates that the RPN and classifier of faster R-CNN used in this study were sufficiently trained. 

#### 5.3.2. Eye Detection Accuracy Obtained Using the Proposed Method

**Figure 18:**
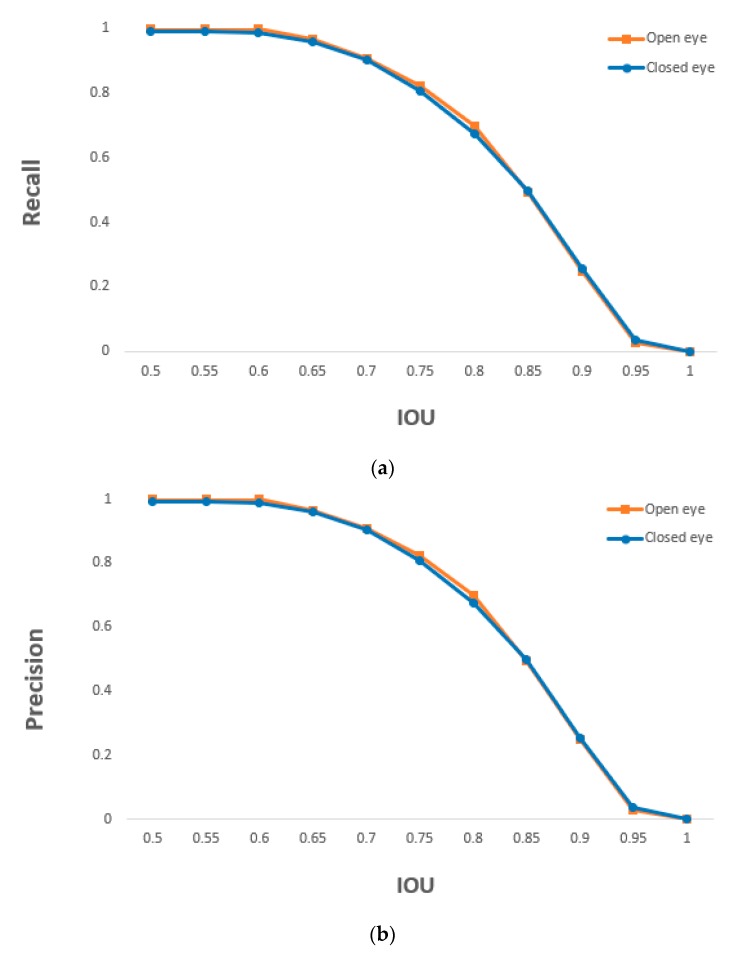
Graphs of (**a**) recall and (**b**) precision of the detection of open and closed eye according to IOU thresholds.

Eyes were detected in the driver images obtained through the faster R-CNN trained in Section 5.3.1. As explained in Section 4.5, geometric transformation was used to map the eye region of the driver images from the two camera images, thereby detecting eye regions in the driver images obtained from both cameras. In addition, by classifying the detected eyes as open eye and closed eye, we obtained additional information on the driver’s eye state, which could be used later to measure inattentiveness. These performance measurements are shown as the average value of the two-fold cross validation. Analysis on recall and precision at different intersection over union (IOU) was used as the standard method for evaluating the existing object detection accuracy. Based on this definition, true positive (TP) indicates that the IOU value of the detected box is greater than the reference IOU threshold and the predicted class matches the annotated information. False positive (FP) indicates that the IOU value of the detected box is smaller than the reference IOU threshold or that the predicted class does not match the annotated information. False negative (FN) indicates that, even if the object is in the image, there is no detected box. At an IOU value of 0.5, Table 8 shows recall and precision for open and closed eye. Based on the aforementioned TP, FP, and FN, we used the following two criteria for accuracy measurements [50]:

(7) $$\mathrm{Precision}=\frac{\#\mathrm{TP}}{\#\mathrm{TP}+\#\mathrm{FP}}$$

(8) $$\mathrm{Recall}=\frac{\#\mathrm{TP}}{\#\mathrm{TP}+\#\mathrm{FN}}$$

where #TP, #FP, and #FN indicate the numbers of TP, FP, and FN, respectively. The minimum and maximum values of precision and recall are 0 and 1, respectively, where 0 and 1 represent the lowest and highest accuracies, respectively. As shown in Table 8, based on the proposed method, open and closed eyes were detected with an accuracy of at least 0.99. Figure 18 shows the recall and precision values according to changes in the IOU threshold. The detection accuracy of open and closed eyes was nearly similar in the entire IOU threshold. 

**Figure 19:**
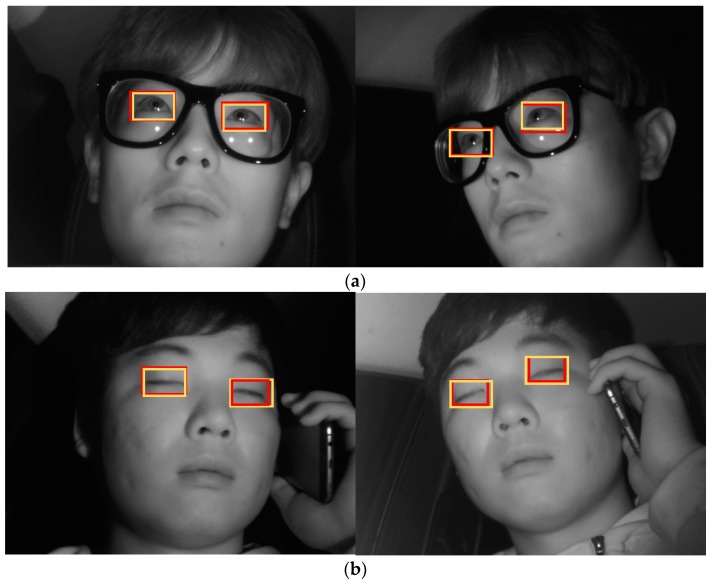
Examples of correctly detected driver’s eye obtained using the proposed method: (**a**) open eye; (**b**) closed eye. (Yellow and red rectangles show the ground-truth and detected boxes, respectively).

**Figure 20:**
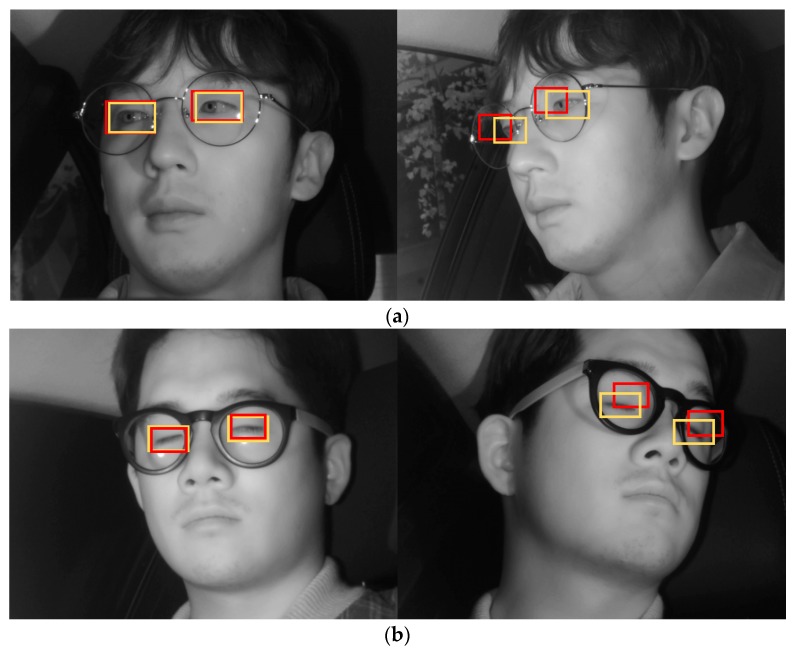
Examples of incorrectly detected eyes obtained using the proposed method: (**a**) open eye; (**b**) closed eye. (Yellow and red rectangles show the ground-truth and detected boxes, respectively).

Figure 19 shows the final correctly detected driver eye detection images obtained through the proposed method, whereas Figure 20 shows the error cases. For the error cases, while the driver’s eyes in frontal face image were accurately detected by faster R-CNN (left images of Figure 20a,b), in the process of mapping the driver eye regions in the side face image through geometric transformation, the regions could not be accurately mapped owing to excessive head rotation (right images of Figure 20a,b).

#### 5.3.3. Comparison of Proposed and Previous Methods on Eye Detection

In this section, we compare the accuracy and processing time of other methods with those of our proposed method for driver’s eyes detection. Other methods include the two faster R-CNNs method in which the frontal face image from shallow CNN is not classified and faster R-CNN is applied for each of two camera images, the AdaBoost eye detection algorithm [29], and the you only look once (YOLO) (versions 2 and 3) detector [51,52]. At an IOU value of 0.5, Table 9 shows that the recall and precision of the proposed method were better than those of the other methods. 

The performance of the two faster R-CNNs method was superior to those of AdaBoost algorithm and YOLO v2 and v3. However, YOLO v2 and v3, which used a one stage method, had faster processing speed than the two faster R-CNNs method. To overcome the problem of slower processing speed compared with that of the YOLO v2 and v3 method, we apply shallow CNN and geometric transformation to the method using one faster R-CNN to maintain a high performance while reducing processing speed. 

**Figure 21:**
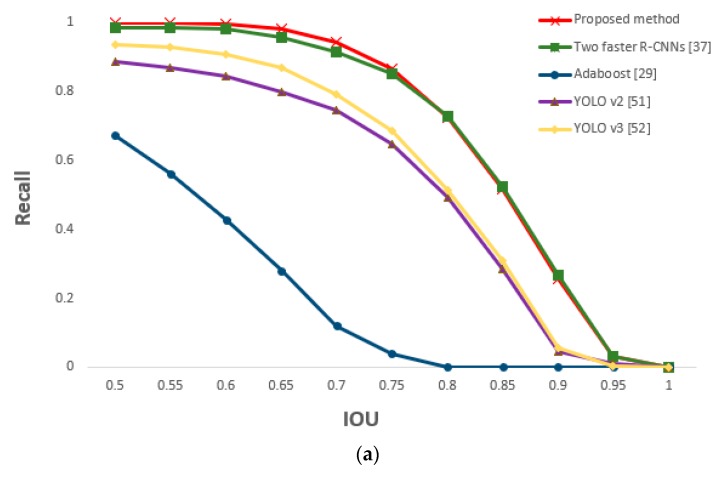

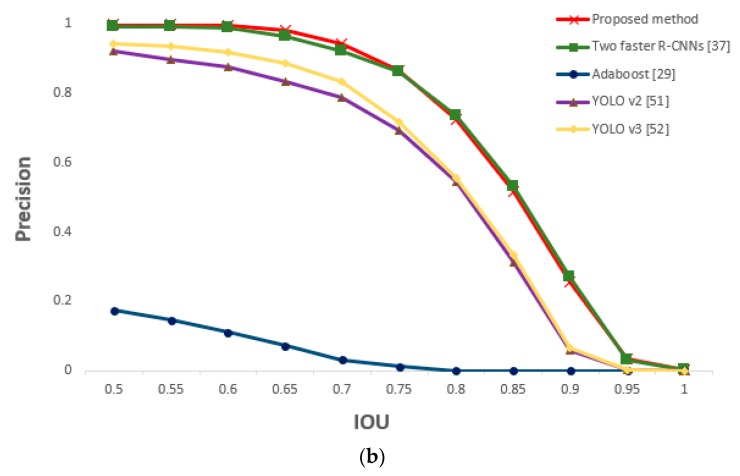
Comparative graphs of (**a**) recall and (**b**) precision of the proposed and previous methods according to IOU thresholds.

For the driver images obtained from the two cameras, faster R-CNN was applied to both in order to detect the eyes. Similar performance as that of the two faster R-CNNs method was obtained; this is because our proposed method is based on faster R-CNN. However, the method of this study is slightly more accurate because it detected the eyes by applying the faster R-CNN only to the frontal face image selected through shallow CNN (easier eye detection). As geometric transformation was applied to the remaining image (i.e., side face image) to map the eyes, the processing time to detect the eyes of both images could be reduced to approximately half of that required in the two faster R-CNNs method. Figure 21 is a graph of the measured recall and precision values according to changes in the IOU threshold, confirming the superior performance of the proposed method.

### 5.4. Performance Evaluations with Open Database

#### 5.4.1. Classification Accuracy of Frontal Face Image Using Shallow CNN

**Figure 22:**
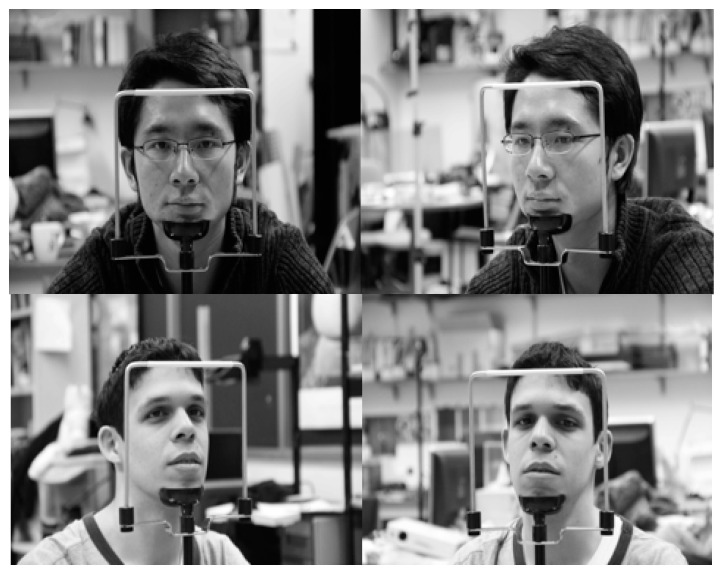
Various head pose image pairs selected from CAVE-DB.

In the next experiment, we used the Columbia Gaze Data Set (CAVE-DB) open database [53] to evaluate the performance of the method proposed in this study. As described in Section 5.1, as this is not an open database of driver’s faces obtained through two cameras in vehicle environments, while not an actual vehicle environment, this study used the CAVE-DB, which acquired facial data of various gaze directions from multiple cameras indoors. This dataset consists of 5880 images of 56 people of various head poses and gaze directions. Among the images of five different head poses per person, we selected one pair of images most similar to the head pose obtained from the two cameras inside the vehicle used in this study. The examples of image pairs with head poses are shown in Figure 22.

We used the same method used in Section 5.2 to obtain augmented data and conduct shallow CNN training. Frontal face image classification accuracy was measured through the trained CNN classifier, the results of which are shown in Table 10. Two-fold cross-validation was used in the accuracy measurement method as well, and the images used in testing were not augmented but were the original ones. As described in Section 5.2.1, for the ground-truth frontal face image which has relatively fewer loss of eye area between two camera images, the image closer to the frontal face was manually selected. If both images had visible eyes and small head rotation and were close to the frontal face, then the image with smaller body rotation was classified as the frontal face image.

**Figure 23:**
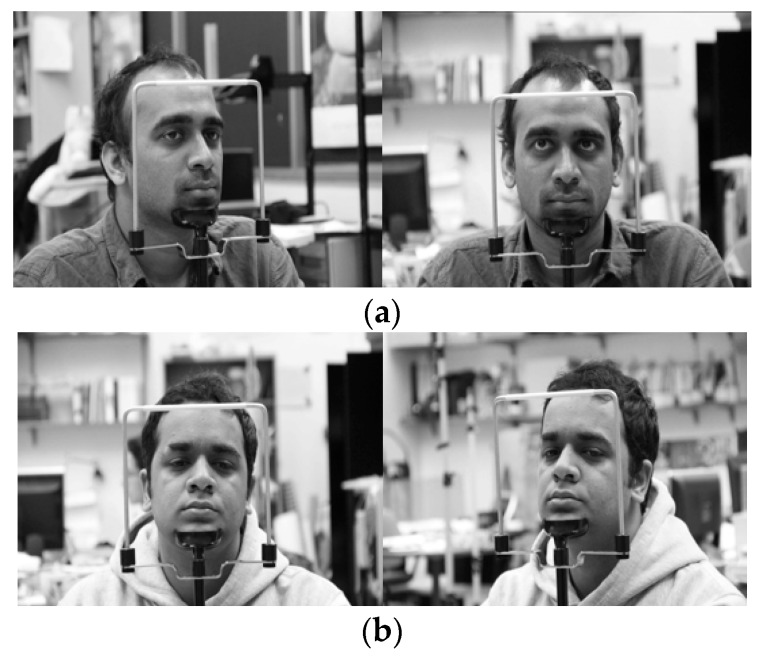
Examples of correct classification of frontal face image. (**a**) Right image selected; (**b**) Left image selected.

**Figure 24:**
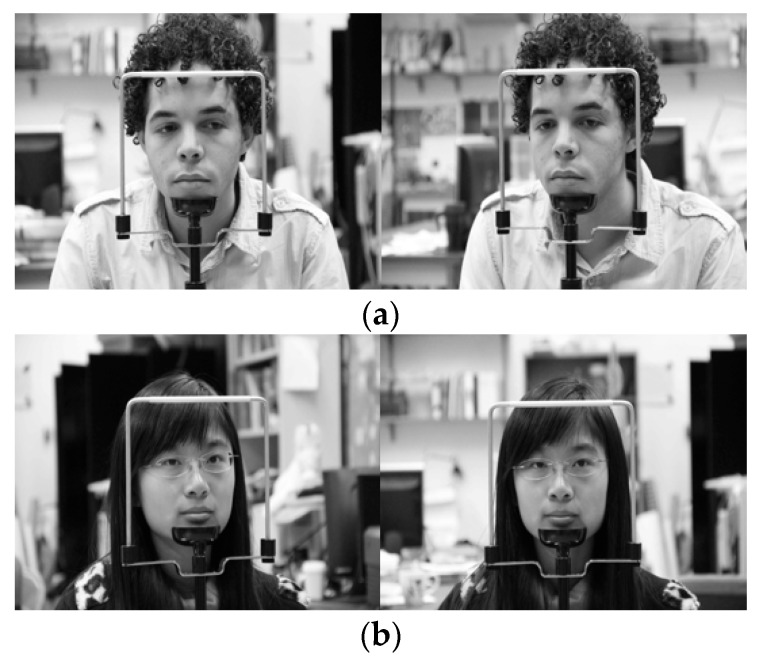
Examples of incorrect classification of frontal face image. (**a**) Right image selected; (**b**) Left image selected.

As shown in Table 10, with an accuracy of at least approximately 99%, the frontal face image was confirmed to be accurately classified based on the method proposed in this study. This is similar to the accuracy of Table 6, indicating that the proposed method can be applied in vehicles and various other environments. Figure 23 shows the correctly classified cases, whereas Figure 24 shows the error cases. In the case of Figure 24, while the classification results seemed incorrect, both images were judged to be close to the frontal face; therefore, using them as the input images of faster R-CNN for the final eye detection is appropriate and would not have a significant effect on the eye detection performance. 

#### 5.4.2. Comparisons of Proposed and Previous Methods on Eye Detection

**Figure 25:**
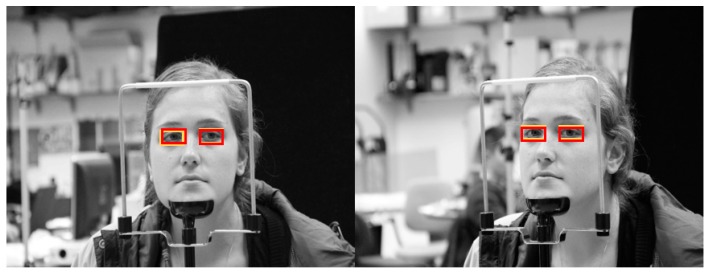
Example of correctly detected eyes by proposed method (Yellow and red rectangles show the ground-truth and detected boxes, respectively).

**Figure 26:**
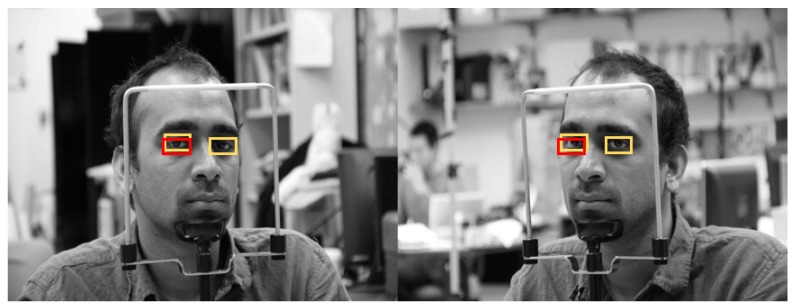
Example of incorrectly detected eye by proposed method (Yellow and red rectangles show the ground-truth and detected boxes, respectively).

**Figure 27:**
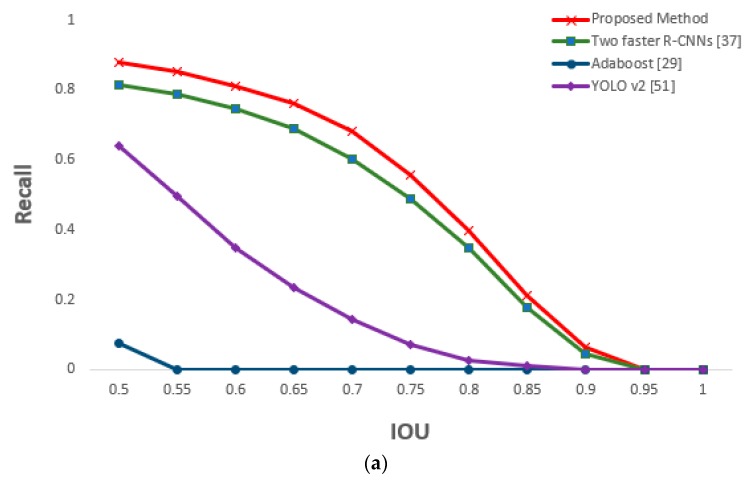

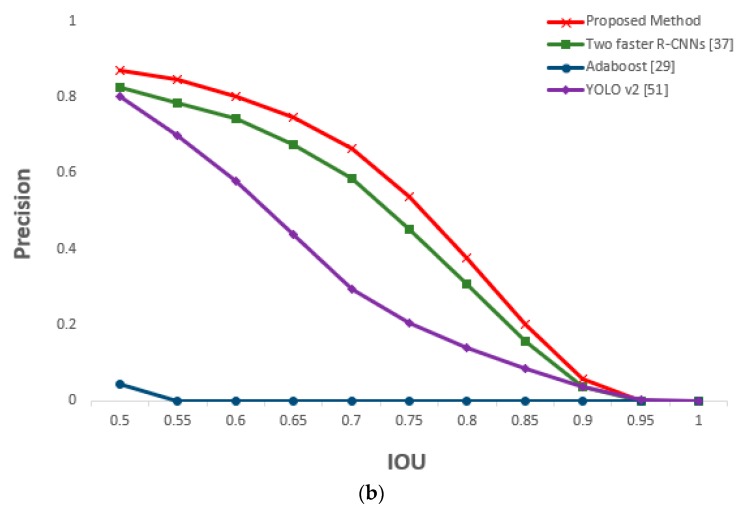
Comparative graphs of (**a**) recall and (**b**) precision of the proposed and previous methods according to IOU thresholds.

For the next experiment, eye detection performance using CAVE-DB was measured. We used the same method used in Section 5.3 to obtain augmented data and conduct faster R-CNN training. Owing to the nature of the open database, we did not conduct closed eye classification and only recorded the performance for open eye detection. All the results are shown through the absolute value of the two-fold cross validation. The augmented data were used only in training, whereas original data were used in testing. The same methods from Section 5.3 were used, and a performance comparison was performed. At an IOU value of 0.5, Table 11 shows that the recall and precision of the proposed method were better than those of the other methods. It is relatively lower than the accuracy measured in Table 9 because the eyes in the CAVE-DB images are significantly smaller than those of the driver images obtained in an actual vehicle environment (DDCD-DB1). Moreover, compared with the existing images, there were more elements to cause erroneous background detections, lowering the accuracy, overall. However, the proposed method showed higher accuracy than the other methods, and processing time could be reduced by approximately half that of the two faster R-CNNs method. Figure 25 shows the final correctly detected eye detection images obtained through the proposed method, whereas Figure 26 shows the error cases. For the error cases, the features of the eye in the image was not correctly detected, so that only one eye of the two eyes of the frontal face image was detected through faster R-CNN, which caused the consequent false negative errors in side face image, also. Figure 27 shows the measured recall and precision values according to the IOU threshold; the accuracy of the proposed method was the highest for all IOU thresholds.

## 6. Conclusions

In this study, we proposed a method of driver’s eye detection in a vehicle environment based on faster R-CNN and geometric transform mapping. While existing methods showed vulnerability to driver head rotation and movement, our method uses a multiple-camera-based method to remain effective even in cases of driver movement and head rotation. Faster R-CNN, a CNN-based object detector, was applied to the driver images obtained through this method to detect the driver’s eyes. The problem of excessive computational cost in the multiple-camera-based method was solved by using shallow CNN and geometric transformation. Through the experiment with our own database, we confirmed the high detection rate of the proposed method and its superior performance compared with those of other detection methods. In addition, evaluations were performed on the open CAVE-DB; the proposed method was confirmed to have better performance.

In conventional researches on pose detection, various poses such as frontal, a little rotated, severely rotated, and side faces, etc. with rotation angles are considered to be predicted. To take this approach in our method, the number of output nodes in the CNN of Figure 4 should be increased according the number of predicted poses, which also increases the training and processing complexities in our CNN. Even in the case of using one output node with the regression scheme in the CNN, the training and processing complexities is also increased. In our research, two images are captured from two cameras as shown in Figure 2 and Figure 3, and complicated pose estimation is not necessary. Our method has only to select one image which is more suitable for the eye detection by faster R-CNN, and we use the CNN having only two outputs as shown in Figure 4.

In future work, we would predict various poses from the input image based on more sophisticated CNN, and use this information in order to understand the detail behaviors and emotion of driver. Although our DDCD-DB1 was collected from 26 people, the number of people is not sufficient. We would plan to extend the dataset collection by including a greater number of subjects in the future. In addition, with the method proposed in this study, we plan to study a driver inattention judgment and emotion recognition system by utilizing open and closed eyes information. And, we would conduct a study on gaze tracking that predicts the driver’s gaze direction based on information about the driver’s eyes detected using multiple cameras.

## Figures and Tables

**Table 1:** Comparison between existing methods and the proposed method for eye detection in vehicle environment.

Category	Methods		Advantage		Disadvantage
Single-camera-based	A single camera is used to detect driver’s eye region [12,13,14,15,16,17]		Relatively faster processing speed owing to low computational cost compared with the multiple-camera-based method		- Vulnerable to the driver’s head movements and rotations
					- Low detection reliability owing to the use of image information from only one camera
Multiple-camera-based	Non-training-based method	Using commercial eye tracker [18,19,20]	- High detection reliability achieved by combining image information from two cameras - Driver allowed to freely move his head - Provides relatively high image resolution even when the driver moves his head	Possible to measure gaze direction through eye detection	Use of expensive commercial eye-tracker
		Facial-feature-points-based method [22,23]		Possible to simply detect eye region during facial feature point detection	Additional process of face detection or facial landmarks detection is required
		Bright-and-dark pupil-based method [24,26]		- Possible to detect eyes in various environments with nearby light using NIR illuminator	- Eye detection is difficult if movements are severe, glasses are worn, or distance from camera is far
				- Faster eye detection possible through simple computation process	- NIR illuminator device is large, making application in actual vehicle environment difficult
	Training-based method	Hough transform and neural network [27]		- Effective eye validation through training method	- Large amount of data required for training
					- Experiment conducted for only the front camera of the two cameras - As the training-based method is used only in detected eye validation, improvement of detection performance is limited
		Faster R-CNN & geometric-transformation-based method (**proposed method**)		- Miniature NIR camera and illuminator used to detect eyes in various nearby lighting conditions	- Large amount of data and time used for training of faster R-CNN
				- Improves processing speed by solving the problem of high computational cost in multiple-camera-based method	
				- Superior eye detection performance through the application of CNN-based method	

**Table 2:** Architecture of feature extractor in Figure 5 (All the convolutional layers (CLs) include ReLU layers).

Layer Type	Number of Filters	Size of Output (Height × Width × Channel)	Size of Kernel	Number of Strides	Number of Paddings
Input layer		800 × 1400 × 3			
1_1st CL	64	800 × 1400 × 64	3 × 3 × 3	1 × 1	1 × 1
1_2nd CL	64	800 × 1400 × 64	3 × 3 × 64	1 × 1	1 × 1
Max pooling layer	1	400 × 700 × 64	2 × 2 × 1	2 × 2	0 × 0
2_1st CL	128	400 × 700 × 128	3 × 3 × 64	1 × 1	1 × 1
2_2nd CL	128	400 × 700 × 128	3 × 3 × 128	1 × 1	1 × 1
Max pooling layer	1	200 × 350 × 128	2 × 2 × 1	2 × 2	0 × 0
3_1st CL	256	200 × 350 × 256	3 × 3 × 128	1 × 1	1 × 1
3_2nd CL	256	200 × 350 × 256	3 × 3 × 256	1 × 1	1 × 1
3_3rd CL	256	200 × 350 × 256	3 × 3 × 256	1 × 1	1 × 1
Max pooling layer	1	100 × 175 × 256	2 × 2 × 1	2 × 2	0 × 0
4_1st CL	512	100 × 175 × 512	3 × 3 × 256	1 × 1	1 × 1
4_2nd CL	512	100 × 175 × 512	3 × 3 × 512	1 × 1	1 × 1
4_3rd CL	512	100 × 175 × 512	3 × 3 × 512	1 × 1	1 × 1
Max pooling layer	1	50 × 88 × 512	2 × 2 × 1	2 × 2	0 × 1
5_1st CL	512	50 × 88 × 512	3 × 3 × 512	1 × 1	1 × 1
5_2nd CL	512	50 × 88 × 512	3 × 3 × 512	1 × 1	1 × 1
5_3rd CL	512	50 × 88 × 512	3 × 3 × 512	1 × 1	1 × 1

**Table 3:** Architecture of RPN in Figure 5 (CL indicates convolutional layer).

Layer Type	Number of Filters	Size of Output	Size of Kernel	Number of Strides	Number of Paddings
[5_3rd CL] Input layer		50 × 88 × 512			
6th CL (ReLU)	512	50 × 88 × 512	3 × 3 × 512	1 × 1	1 × 1
Classification CL (Softmax)	18	50 × 88 × 18	1 × 1 × 512	1 × 1	0 × 0
[6th CL] Regression CL	36	50 × 88 × 36	1 × 1 × 512	1 × 1	0 × 0

**Table 4:** Architecture of classifier in Figure 5 (ROI coordinate * includes the values of x_min, y_min, x_max, and y_max of ROI for each proposal) (CL and FCL indicate convolutional layer and fully connected layer, respectively.).

Layer Type	Size of Output
[5_3rd CL] [RPN proposal region] Input layer	50 × 88 × 512 (height × width × depth) 300 × 4 (ROI coordinate *)
ROI pooling layer	7 × 7 × 512 (height × width × depth) × 300
1st FCL (ReLU) (Dropout)	4096 × 300
2nd FCL (ReLU) (Dropout)	4096 × 300
Classification FCL (Softmax)	3 × 300
[2nd FCL] Regression FCL	4 × 300

**Table 5:** Image database for the evaluation of shallow CNN.

Two-Fold Cross Validation	Training	Testing
1st fold validation	75,975 (3039 × 25) images from 13 people	3149 images from 13 people
2nd fold validation	78,725 (3149 × 25) images from 13 people	3039 images from 13 people

**Table 6:** Classification accuracies by our shallow CNN (unit: %).

Accuracy	1st Fold Validation	2nd Fold Validation	Average
Our shallow CNN	99.42	100	99.71

**Table 7:** Image database for the evaluation of faster R-CNN.

Two-Fold Cross Validation	Kinds of Images	Training (Augmented Images)
1st fold validation	Open eye images	8974 (4487 × 2)
	Closed eye images	9248 (4624 × 2)
	Total	18,222 (9111 × 2)
2nd fold validation	Open eye images	10,272 (5136 × 2)
	Closed eye images	9004 (4502 × 2)
	Total	19,276 (9638 × 2)

**Table 8:** Recall and precision for the detection of open and closed eye using the proposed method at an IOU threshold of 0.5.

Class	Recall	Precision
Open eye	0.9989	1
Closed eye	0.9906	0.9916

**Table 9:** Comparisons of recall, precision, and processing time of the proposed and previous methods at an IOU threshold of 0.5.

Method	Recall	Precision	Processing Time (Unit: ms)
AdaBoost [29]	0.67	0.17	65
YOLO v2 [51]	0.89	0.92	40
YOLO v3 [52]	0.93	0.94	62
Two faster R-CNNs [37] (without shallow CNN)	0.98	0.99	236
Proposed method	0.99	1	121

**Table 10:** Classification accuracy of frontal face image using shallow CNN (unit: %).

	1st Fold Validation	2nd Fold Validation	Average
Accuracy	99.23	98.87	99.05

**Table 11:** Comparisons of recall, precision, and processing time of the proposed and previous methods at an IOU threshold of 0.5.

Method	Recall	Precision	Processing Time (Unit: ms)
AdaBoost [29]	0.07	0.04	67
YOLO v2 [51]	0.64	0.80	41
Two faster R-CNNs [37] (without shallow CNN)	0.81	0.82	235
Proposed method	0.88	0.87	126
